# Combating Antimicrobial Resistance Through Student-Driven Research and Environmental Surveillance

**DOI:** 10.3389/fmicb.2021.577821

**Published:** 2021-01-22

**Authors:** Erica R. Fuhrmeister, Jennifer R. Larson, Adam J. Kleinschmit, James E. Kirby, Amy J. Pickering, Carol A. Bascom-Slack

**Affiliations:** ^1^Department of Civil and Environmental Engineering, Tufts University School of Engineering, Medford, MA, United States; ^2^Department of Biological and Environmental Sciences, Capital University, Columbus, OH, United States; ^3^Department of Natural and Applied Sciences, University of Dubuque, Dubuque, IA, United States; ^4^Department of Pathology, Beth Israel Deaconess Medical Center, Boston, MA, United States; ^5^Department of Medical Education, Tufts University School of Medicine, Boston, MA, United States

**Keywords:** antimicrobial resistance, antibiotic resistance, environmental surveillance, One Health, science education, citizen science, CURE (course-based undergraduate research experience)

## Abstract

Emerging resistance to all classes of antimicrobials is one of the defining crises of the 21st century. Many advances in modern medicine, such as routine surgeries, are predicated on sustaining patients with antimicrobials during a period when their immune systems alone cannot clear infection. The development of new antimicrobials has not kept pace with the antimicrobial resistance (AR) threat. AR bacteria have been documented in various environments, such as drinking and surface water, food, sewage, and soil, yet surveillance and sampling has largely been from infected patients. The prevalence and diversity of AR bacteria in the environment, and the risks they pose to humans are not well understood. There is consensus that environmental surveillance is an important first step in forecasting and targeting efforts to prevent spread and transmission of AR microbes. However, efforts to date have been limited. The Prevalence of Antibiotic Resistance in the Environment (PARE) is a classroom-based project that engages students around the globe in systematic environmental AR surveillance with the goal of identifying areas where prevalence is high. The format of PARE, designed as short classroom research modules, lowers common barriers for institutional participation in course-based research. PARE brings real-world microbiology into the classroom by educating students about the pressing public health issue of AR, while empowering them to be partners in the solution. In turn, the PARE project provides impactful data to inform our understanding of the spread of AR in the environment through global real-time surveillance.

## Introduction

In October, 2020, the new United States National Action Plan for Combating Antibiotic-Resistant Bacteria 2020–2025 was published (Federal Task Force on Combating Antibiotic-Resistant Bacteria, 2020). This plan builds on the roadmap laid out in 2015, which takes a comprehensive approach to addressing the worldwide problem of antimicrobial resistance (AR). The approach is rooted in the concept of One Health, which recognizes that human health is inextricably linked to the environment, other animals, and plants (Zinsstag et al., 2011). Since then, coordinated progress has been made in several areas, yet one national goal with enormous untapped potential is that of strengthening national One Health surveillance efforts.

Surveillance of AR in the United States has focused mainly on food testing and epidemiological tracking of clinical infection (Lammie and Highes, 2016). Clinicians and epidemiologists respond primarily to existing threats. Patients present with a resistant infection before epidemiologists are invoked to trace the source and contain the spread. Epidemiological tracking of infectious disease can contain transmission and food testing is a proactive, preventative measure, but more must be done to prevent infections before they arise. As a result of the SARS-CoV-2 pandemic, there is increased awareness of the need to study microbes and transmission patterns in non-clinical settings (i.e., the environment) to prevent the emergence of infectious disease outbreaks.

Antibiotics discharged into the environment provide a selective pressure for survival of microbes harboring AR. Waste streams from livestock, hospitals, households, and industry production of antibiotics can contribute to dissemination of antibiotics and AR into the environment, and AR bacteria and AR genes have been documented in numerous environmental reservoirs (e.g., Aarestrup et al., 1998; Nwosu, 2001; Schwartz et al., 2003; D’Costa et al., 2006; Salyers and Shoemaker, 2006; Lobova et al., 2008; Allen et al., 2010; Institute of Medicine (US) Forum on Microbial Threats, 2010; Wright, 2010; Berglund, 2015; Li et al., 2015). Robust environmental surveillance of water and soil samples has the potential to identify hotspots of AR, which could lead to localized stewardship efforts to contain spread of resistance, possibly preventing AR outbreaks.

Environmental scientists generally conduct in-depth studies to monitor presence and/or abundance of AR genes or levels of culturable AR bacteria in environmental samples from a limited geographic range, but we argue that a more proactive and coordinated approach among clinicians, epidemiologists, and environmental scientists must be taken. For example, there remains a disconnect between the resistance genes surveyed in the environment and those deemed clinically relevant by medical practitioners. Even among scientists, groups rarely use the same sampling schemes, methods, or reporting metrics, making comparisons across studies challenging. We propose that this gap can be addressed, in part, by undergraduates as citizen scientists conducting environmental surveillance to identify environmental hotspots of AR, with findings made available in a database. At the same time, engaging undergraduates in a large research study has the potential to fill an unmet need in undergraduate education – providing more access to authentic research experiences.

Despite the environmental presence of AR bacteria and widespread use of antimicrobials, the public is generally unaware of environmental resistance as a global public health problem (McCullough et al., 2016; Wellcome Trust, 2019). This is compounded by lack of understanding regarding natural selection and how presence of antibiotics leads to selection for AR bacteria. Reduction in use of antimicrobials in agriculture, aquaculture, veterinary, and human medicine are all critical to decrease selective pressure for survival and spread of resistant microbes. One notable example of the power of the informed public is the increase in consumer demand for meat production without antibiotics, which has driven food chains to seek out sources of animals treated with fewer or no antibiotics (Neilsen FreshFacts, 2016; Halloran and Bohne, 2017; Singer et al., 2019). This is significant because interventions that restrict antibiotic use in food animals are tightly associated with a decrease in AR bacteria in these animals (Tang et al., 2017). Collectively, an educated public and the resulting behavioral changes have the power to impact the issue of AR through reducing unnecessary use of antimicrobials.

Currently there is a need for consistent messaging that engages citizens in antibiotic stewardship. Educating the public about the growing AR crisis has been identified in the President’s Council of Advisors on Science and Technology (PCAST) *Report on Combating Antibiotic Resistance* as a critical factor for improving antimicrobial stewardship, especially regarding the demand for inappropriate antimicrobial prescriptions (Olson and Riordan, 2012). In addition, many organizations including the [World Health Organization (WHO), 2015], the Wellcome Trust (2019), and the Centers for Disease Control and Prevention (Federal Task Force on Combating Antibiotic-Resistant Bacteria, 2020) have stated that educating the general public is a critical component in the battle against AR. We argue that the classroom is an ideal environment, that of a captive audience, in which to convey consistent, but simple messaging regarding the issue of AR and actions that can be taken by individuals.

## Course-Based Research Experiences: Improved Pedagogy and Increased Scientific Potential

The last 70 years have brought a revolutionary shift in the way we approach teaching laboratory courses. Inquiry-based learning aims to move from the traditional memorization of facts to a process that allows students to “discover” scientific principles through their own experimentation (National Research Council, 1996, 2000; Chiappetta, 2007). Course-based undergraduate research experiences (CUREs) could be considered the next generation of inquiry-based learning. In contrast to inquiry-based instruction, in which the results are generally of limited interest to the broader scientific community, CUREs provide an opportunity for students (typically undergraduates) to collect and analyze data that have potential to lead to new scientific findings of interest beyond the classroom (Lopatto, 2003; Hatfull et al., 2006; Buck et al., 2008; Kloser et al., 2011; Fukami, 2013; Alaimo et al., 2014; Auchincloss et al., 2014; Spell et al., 2014; National Academies of Sciences, Engineering, and Medicine, 2015; Weaver et al., 2020) and are an attempt to scale up the traditional apprentice-style research experiences for undergraduates (Wei and Woodin, 2011; Olson and Riordan, 2012; National Academies of Sciences, Engineering, and Medicine, 2015). Placing the research experience in the context of a classroom provides opportunities for students who may not otherwise have access to research, such as those attending community colleges or who cannot afford to engage in an unpaid, out-of-class program or internship (Bangera and Brownell, 2014; Hensel and Davidson, 2018).

There is growing recognition that authentic research experiences are valuable to promote desired outcomes in early STEM education, especially retention, improvement in academic achievement, and matriculation into graduate and professional programs. Recently, there has been an explosion in interest in and development of CUREs (e.g., American Association for the Advancement of Science, 2011; Wei and Woodin, 2011; Olson and Riordan, 2012; National Academies of Sciences, Engineering, and Medicine, 2015). In addition to the valuable potential of CUREs to engage and retain STEM students, CUREs have proven effective tools to engage students in tackling scientific challenges that are not well suited to an individual research group, but instead require a large cohort to work en masse.

A key aspect to many successful CUREs is the power of contributing to a collective data set that is available to the larger research community (Hatfull et al., 2006; Shaffer et al., 2010; Jordan et al., 2014; Wiley and Stover, 2014; Leung et al., 2015). Programs with early success in crowd-sourcing student-generated data to tackle big research questions have provided inspiration for scientists in other fields to consider harnessing the power of undergraduates in the classroom to advance science (Elgin et al., 2016, 2017). Students participating in course-based research have contributed to scientific knowledge both through peer-reviewed publications and contributions to databases (e.g., Jordan et al., 2014; Leung et al., 2015; Elgin et al., 2017). The power of student-generated discoveries has been further solidified with the recent publication describing treatment of a cystic fibrosis patient with a combination of engineered, student-derived bacteriophages that killed an infectious strain of *Mycobacterium abscessus* (Dedrick et al., 2019).

## The Prevalence of Antibiotic Resistance in the Environment: A Classroom-Based Environmental Surveillance Project

**Figure 1:**
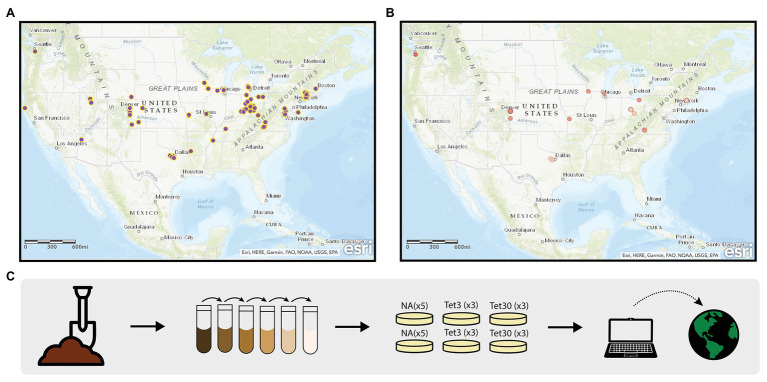
Prevalence of Antibiotic Resistance in the Environment (PARE) Project. The geographic information systems (GIS)-based maps depicting student soil sample collection sites **(A)**, and samples harboring culturable bacteria resistant to 30 μg/ml tetracycline **(B)**. Dark red dots represent samples in which greater than 25% of colony forming units were resistant; light red dots represent less than 25% resistance. **(C)** Outlines the methods used by students to obtain the data shown in the maps. Detailed description of methods available in Genné-Bacon and Bascom-Slack (2018).

The Prevalence of Antibiotic Resistance in the Environment (PARE) project is a powerful platform for student learning and scientific discovery that has potential to address the scientific challenges associated with environmental surveillance of AR and to educate tomorrow’s decision makers about the public health threat of AR and the biological concept of natural selection. The PARE project is a CURE in which standardized student crowdsourcing data are used to generate a Geographic Information Systems (GIS)-based map of AR data (Figures 1A,B). The overarching scientific goal is to identify environmental hotspots of AR that could present risk for human exposure and infection. The educational goal is to make research more equitable for students by creating a scalable, sustainable research program that can be carried out in classrooms at the undergraduate level, while extending across institution types.

The reach of PARE transcends traditional cohorts of undergraduate biology majors as the curriculum is uniquely situated to engage heterogeneous groups of students beyond the microbiology classroom including non-biology majors and high school students. PARE provides a captivating context for teaching a wide array of core interconnected biological principles (e.g., natural selection, adaptation, mutagenesis, and cell structure/function), while addressing the contemporary public health issue of AR. Students experience and learn about the process of science through the design and execution of experiments, and through grappling with the messiness inherent in authentic experimental data.

In the original, core PARE module (Genné-Bacon and Bascom-Slack, 2018) students collect soil, perform serial dilutions, and plate onto media with or without defined concentrations of tetracycline (Figure 1C). Tetracycline was selected for this surveillance project, in part, because it is inexpensive, easy to use and it has a long history of use and study in agriculture (Daghrir and Drogui, 2013; Zhou et al., 2017). Students analyze growth on plates, then calculate and upload the prevalence of tetracycline-resistant colony forming units into a database. Once students upload their PARE results, their prevalence data appears instantly with their soil collection site on an interactive, web-based map. Feedback from participating faculty indicates that a motivator for participating in PARE is the knowledge that students are part of a research community, contributing to a larger body of knowledge (Genné-Bacon et al., 2020).

## Data Curation

Curation of student-generated data has been a challenge; yet, we have created a new data upload and display system to address many issues that became apparent after the first few years of data collection. Our current system allows display of all soil data collection sites, but blocks display of tetracycline resistance data that do not meet curation criteria. This new GIS-based system was introduced just prior to the onset of the COVID-19 pandemic in which most instructors had to abort their in-person teaching. Nonetheless, this fall, nearly 300 student entries have been recorded. Students upload global positioning system (GPS) metadata captured from a smartphone at the time and place of their sample collection. This eliminates a previous problem of inaccurate location information. Another common observed problem is errors in calculations. For determination of prevalence data, students must count colonies that grow on media with and without tetracycline, and then perform calculations to determine the percent of colonies that are resistant. Analyses of earlier datasets indicated that about 1/3 of entries contained calculation errors. Error types included incorrect use of scientific notation, (e.g., incorrect by an order of magnitude), simple arithmetic errors (e.g., failure to account for dilution factors), and missing data. In our current automated curation, prompts are designed to eliminate scientific notation errors and each step that requires a calculation is calculated automatically based on student colony counts entered. If the computer-generated value does not equal student-generated value, a warning message appears notifying the student and providing opportunity for them to re-check their entries and calculations. Our curation standard for tetracycline resistance data display also requires entry of two duplicate plate sets from the same soil sample. These duplicate counts must not be more than 50% different. These measures have solved many previous problems with the data quality. We cannot, however, verify that students perform accurate colony counts, although two students are instructed to first independently count then compare and arrive at consensus. In addition, depending on the local environment, some dilution sets are simply not countable due to contamination, overgrowth, or undergrowth. To date, of 294 entries, 20% pass our curation standard. While this number is low, we believe, we can reach significant numbers because the growing network of participants implementing PARE includes thousands of students at over 120 undergraduate institutions, approximately 20 high schools, and international partners in France, India, and Botswana. We believe this “first pass” of surveillance complements the rigorous in-depth, but geographically-limited studies being conducted by environmental scientists.

## Data Analysis

Students are asked to form a hypothesis about expected levels of AR in their samples based on collection site characteristics. After resulting AR frequencies are calculated, students first enter data into a shared cloud-based spreadsheet that provides an opportunity to view the classroom data, and to identify and correct errors before database upload. A curated, comprehensive spreadsheet of data is also available on our website for instructors who prefer to have their students work with a larger dataset. In analyses of the data, students are confronted with the messiness of real biological data, which serves as an important learning opportunity and puts them in the shoes of scientists. Students have reported that this reflection helps reinforce the importance of careful attention to methods. Upon reflecting on classroom results, students are asked whether they are consistent with predictions and engage in discussion of potential explanations. Simple analytical activities include determining how best to represent the classroom data and use of *t*-tests to compare average resistance levels at different sites, both of which help build quantitative skills. Some instructors also require students to produce a scientific poster or oral presentation of their results. The student GIS-based database provides enormous potential for data visualization and analysis. Student data can be mapped and analyzed in relation to existing GIS datasets such as land use patterns, pollution/emissions monitoring, and health tracking. A future goal is to augment the curriculum with instructions for students to carry out these analyses.

## Pare is a Flexible and Inclusive Way to Engage Diverse Students

Approximately one-third of PARE-participating institutions are community colleges. This is notable because there are known barriers associated with faculty development and implementation of CUREs (Spell et al., 2014; Harris et al., 2015; Shortlidge et al., 2016; Craig, 2017), and data suggest that these tend to be particularly significant for faculty at community colleges (Spell et al., 2014). One way in which PARE lowers barriers for implementation is through its modular design. Since its inception, PARE has partnered with instructors, faculty experts, and industry to design a suite of classroom research modules, complementary to the core module (Table 1). The modules integrate a variety of methods (culture-based and molecular) to assay environmental samples for indications of AR bacteria or associated molecular markers. Table 1 lists the modules in order of general progression in terms of technical difficulty, time, and reagents required; however, there is no set order in which the modules must be introduced. This flexibility draws instructors from a wide variety of courses and institution types, many of whom have never introduced authentic research into their laboratory courses before. The modular nature of PARE reduces faculty perceived barriers to adopting CUREs by providing faculty with the ability to transition from a traditional laboratory curriculum to a CURE by progressively adding subsequent research modules (Genné-Bacon et al., 2020). It has been documented that, for students, the benefits gained from CUREs increase with time spent immersed within the research project (Shaffer et al., 2014), so it is notable that the majority of PARE instructors transition from implementation of the core module to an expanded experience by their second year of implementation (unpublished results). PARE is continuing to expand its scientific impact through development of additional modules to assess presence of clinically relevant AR genes such as *bla*_NDM1_, *mcr1*, and ribosomal methylase-mediated aminoglycoside resistance. Future plans include proactively seeking to detect resistance to newly marketed antimicrobials and those with promise in development.

**Table 1:** PARE project laboratory module descriptions.

Module name and type	Description	Major skills embedded	Difficulty
Data analysis case study (*Online possible*)	A classroom exercise created with the Great Diseases project, guiding students through a simplified version of a seminal report investigating the outcome of introducing tetracycline use on farms (Jacque et al., 2013).	Data analysis	Introductory level
Molecular case study	A known-outcome case study narrative, created in collaboration with miniPCR™ (https://www.minipcr.com), in which students employ PCR to trace the source of a simulated antimicrobial resistance (AR) outbreak.	PCR Gel electrophoresis	Introductory level
Core module	A culture-based exercise in which soil samples are diluted and plated onto media with and without tetracycline to determine relative prevalence of AR microbes (152).	Serial dilution Plating CFU determination	Introductory or upper level
Virtual colony count (*Online possible*)	An online activity to simulate the Core PARE module.	CFU determination	Introductory or upper level
Identification of Tc^R^ genes	A non-culture-based activity in which soil DNA is extracted and tested for the presence of two common tetracycline-resistance genes, t*etM* and *tetA* using PCR [created in collaboration with miniPCR™].	DNA extraction PCR Gel electrophoresis	Introductory or upper level
Colony identification	16S rRNA gene sequence analysis is performed on DNA extracted from tetracycline-resistant colonies (isolated in the core module) to make a preliminary phylogenetic assignment.	DNA extraction PCR Gel electrophoresis BLAST search Sequence analysis	Introductory or upper level
Multi-drug resistance testing	Colonies isolated in the core PARE module are tested for resistance to other antimicrobials using a Kirby-Bauer assay.	Culturing Kirby-Bauer testing	Introductory or upper level
Identification of clinically important resistance genes (*in development*)	Soil DNA is extracted and tested for the presence of emergent resistance gene markers (e.g., *bla*_NDM1_ and *mcr1*).	DNA extraction PCR Gel electrophoresis	Introductory or upper level
Bioinformatics 1 (*Online possible*)	A computer-based sequence analysis activity comparing tetracycline-resistance genes from different bacterial species.	Sequence analysis BLAST search	Introductory or upper level
Bioinformatics 2 (*Online*)	Using on online bioinformatics workflow to search metagenomic soil and water DNA sequence for antibiotic resistance genes.	Interpretation and analysis of bioinformatics data	Upper level
Horizontal transfer	Tests for the ability of the resistance determinant to transfer *via* horizontal gene transfer.	Plasmid extraction Bacterial transformation Plating	Upper level

Modules are generally organized from least to most challenging in terms of technical skills and/or content. There is no specific order in which the modules must be completed. CFU, colony forming units; TcR, tetracycline resistance; AR, antimicrobial resistant.

Many science departments are currently working to integrate a scaffolded CURE experience into the curriculum for undergraduates by offering a progression in which skills or concepts learned in an introductory-level CURE are built upon in a later, upper-level CURE. PARE provides an opportunity for scaffolding of different modules in different courses. In addition, it is complementary to other nationally-disseminated semester-long CUREs such as Tiny Earth/Small World Initiative (Barral et al., 2014, 2016; Hurley et al., 2020), SEA PHAGES (Hatfull et al., 2006; Jordan et al., 2014), and Authentic Research Experience in Microbiology (Muth and McEntee, 2014; Muth and Caplan, 2020). All are focused on some aspect of environmental microbiology, so together they provide ready-to-use options for scaffolding. Tiny Earth provides a particularly appealing complement to PARE due to its focus on discovery of antibiotic-producing microbes from soil samples.

## Discussion

A lack of systematic contributions by governmental agencies, non-profits, and research laboratories has left a knowledge deficit that provides an ideal opportunity for PARE to educate students on the problem of environmental resistance and engage them in a research experience with potential for meaningful scientific and societal impact. By engaging a broad constituency of students, including those who will funnel into the healthcare workforce as well as non-STEM sectors of society, PARE directly addresses the need for effective, widely-disseminated messaging to explain the One Health nature of AR and does so in a tangible, personally compelling manner. In addition, the establishment of a standardized environmental surveillance system has the potential to identify hotspots of AR, identify human exposure pathways, monitor for emerging resistant pathogens, and generate data for educational programs to increase public awareness. Increasing understanding of the distribution of AR in their own community not only provides a powerful hook to engage students but also provides an opportunity to enlist them as research partners in a project with broad scientific merit. Students who participate in PARE will further serve as future ambassadors within and outside of healthcare environments, disseminating the critical message that AR is a threat that everyone should not only care about, but one in which they can directly participate in the solution by improving their antimicrobial stewardship.

## Author Contributions

CB-S wrote the majority of the manuscript with contributions on the environmental perspective from EF and AP and on the clinical perspective from JK, and performed the literature search. JL and AK contributed to sections on the Prevalence of Antibiotic Resistance in the Environment Project and public perceptions. EF created the figure. All authors contributed to the article and approved the submitted version.

### Disclaimer

Any opinions, findings, and conclusions or recommendations expressed in this article are those of the authors and do not necessarily reflect the views of the National Science Foundation.

### Conflict of Interest

The authors declare that the research was conducted in the absence of any commercial or financial relationships that could be construed as a potential conflict of interest.
